# Progress in Predicting Ames Test Outcomes from Chemical Structures: An In-Depth Re-Evaluation of Models from the 1st and 2nd Ames/QSAR International Challenge Projects

**DOI:** 10.3390/ijms25031373

**Published:** 2024-01-23

**Authors:** Yoshihiro Uesawa

**Affiliations:** Department of Medical Molecular Informatics, Meiji Pharmaceutical University, Tokyo 204-8588, Japan; uesawa@my-pharm.ac.jp

**Keywords:** Ames test, quantitative structure–activity relationship, applicability domain, in silico study, machine learning, predictive performance

## Abstract

The Ames/quantitative structure–activity relationship (QSAR) International Challenge Projects, held during 2014–2017 and 2020–2022, evaluated the performance of various predictive models. Despite the significant insights gained, the rules allowing participants to select prediction targets introduced ambiguity in model performance evaluation. This reanalysis identified the highest-performing prediction model, assuming a 100% coverage rate (COV) for all prediction target compounds and an estimated performance variation due to changes in COV. All models from both projects were evaluated using balance accuracy (BA), the Matthews correlation coefficient (MCC), the F1 score (F1), and the first principal component (PC1). After normalizing the COV, a correlation analysis with these indicators was conducted, and the evaluation index for all prediction models in terms of the COV was estimated. In total, using 109 models, the model with the highest estimated BA (76.9) at 100% COV was MMI-VOTE1, as reported by Meiji Pharmaceutical University (MPU). The best models for MCC, F1, and PC1 were all MMI-STK1, also reported by MPU. All the models reported by MPU ranked in the top four. MMI-STK1 was estimated to have F1 scores of 59.2, 61.5, and 63.1 at COV levels of 90%, 60%, and 30%, respectively. These findings highlight the current state and potential of the Ames prediction technology.

## 1. Introduction

The Ames test, a biological assay that utilizes bacterial strains such as Salmonella typhimurium, is a widely used method for assessing chemical mutagenicity by monitoring reverse mutations [1]. This test serves as a preliminary screening tool to evaluate the carcinogenic potential of chemicals [2]. Recently, the focus has shifted toward developing new methods for the initial assessment of impurities in pharmaceuticals. The international conference on harmonization (ICH) M7 guideline promotes the use of quantitative structure–activity relationship (QSAR) models as an alternative to traditional toxicological studies [3], making the accuracy of QSAR models in identifying mutagenic chemicals increasingly important.

The Division of Genetics and Mutagenesis at the National Institutes of Health Sciences in Japan (DGM/NIHS) has developed an Ames mutagenicity database, which includes chemicals not previously incorporated in QSAR model development. The Ames/QSAR International Challenge Project [4], conducted between 2014 and 2017, involved twelve QSAR vendors from seven countries and tested seventeen QSAR tools across three distinct phases (i.e., Phases I–III). Phases I, II, and III were performed between 2014 and 2015, 2015 and 2016, and 2016 and 2017 with a total of 3902, 3829, and 4409 compounds, respectively. A total of 12,140 compounds were used as an external validation set, with the Ames test data for these chemicals sourced from unpublished data registered under Japan’s Industrial Safety and Health Act (ANEI-HOU) at the Ministry of Health, Labour and Welfare [5]. However, due to confidentiality concerns, the ANEI-HOU data are not publicly accessible. The compounds were divided into three phases, each with unique implementation periods. Rigorous external validation was conducted using these compounds, and the predictive performance of each QSAR tool was assessed using various metrics. The external validation set compounds and correct answers from the previous phase were disclosed to the participants, leading to a significant improvement in the predictive abilities of almost all QSAR tools, particularly in Phase II, compared with Phase I. In a continued effort to enhance QSAR model development, the DGM/NIHS orchestrated the second Ames/QSAR International Challenge Project from 2020 to 2022 [6]. This iteration utilized unpublished Ames test results of 1589 novel chemical substances, based on ANEI-HOU, as the test dataset. The rules were akin to those of the inaugural project. Furthermore, participants were supplied with Ames test data for the 12,134 compounds, curated post the first round, along with corresponding SMILES notations in SDF files, to serve as a training set. The teams had the liberty to utilize this training data and all known data for model construction. The challenge witnessed the participation of 21 teams, encompassing academic institutions from 11 countries. Each team employed a variety of Ames/QSAR models to predict the mutagenicity of test chemicals. Performance comparisons were made with the first project, revealing that the results from the second round were generally not as robust. However, teams that participated in both rounds demonstrated commendable average results. In both projects, the rules allowed models to select target compounds for prediction arbitrarily, which introduced ambiguity into the performance evaluation. Furthermore, neither project report examined the impact of coverage ratio (COV) variation on prediction performance. It is well-established that defining the predicted chemical space based on an appropriate applicability domain significantly influences the generalization performance of QSAR models [7,8,9,10]. Therefore, the methodology for determining COV, a factor distinct from the model’s inherent performance, becomes critical. Given the flexibility in setting the COV, it is conceivable that models with lower COVs demonstrated superior predictive performance. To accurately evaluate the current state of Ames test QSAR analysis, it is crucial to distinguish between the model construction technology and the appropriate COV setting. As a result, this paper first reanalyzes all models from the first and second projects to ascertain the impact of COV on prediction performance. Subsequently, I aim to identify the highest-performing prediction model at a standard 100% COV, taking into account COV effects. Additionally, I estimate how the generalization performance of the optimal model fluctuates with standard COV shifts determined from all models.

## 2. Results

### 2.1. Data for the Analysis

This study evaluated a total of 109 models, which included 58 models from the first project and 51 models from the second project. These models were derived from previously reported papers [4,6], inclusive of those detailed in the supplementary data. The projects saw participation from 27 teams, with some teams participating in both projects. Each team developed between one and fourteen types of models (Table S1). It is important to note that even if a model name was repeated across different phases of each project, it was considered an independent model. To distinguish these models, they were assigned unique serial numbers.

### 2.2. Evaluation Index

**Figure 1:**
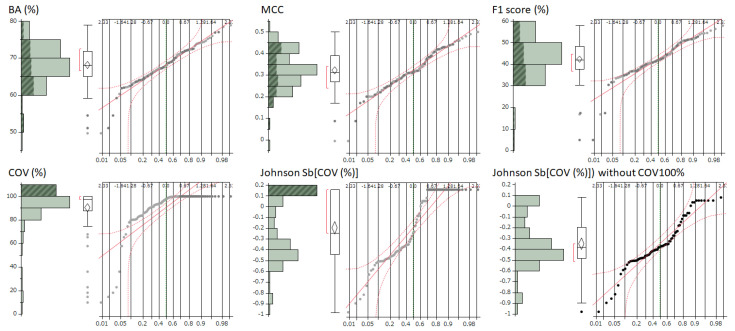
Shape of the performance evaluation index and coverage rate distribution for predictive models. The terms BA (%), MCC, COV (%), Johnson Sb[COV (%)], and Johnson Sb[COV (%)] without COV 100% represent various evaluation metrics for the prediction models. Specifically, BA (%) refers to the balanced accuracy, MCC stands for Matthews correlation coefficient, and COV (%) denotes the coverage rate for all predicted target compounds. Johnson Sb[COV (%)] represents the Johnson normalized coverage rate, while Johnson Sb[COV (%)] without COV 100% signifies the Johnson normalized coverage rate, excluding the prediction models with a 100% coverage rate. Prediction models that had 100% coverage are highlighted in dark. Out of the 109 types of prediction models evaluated in this study, 33 models had a coverage rate of 100%. These normal quantile plots are shown as dots and red lines.

We examined the distribution of evaluation indicators for the prediction models, BA (%), MCC, F1 (%), and COV (%), using normal quantile plots. All the indicators except COV fell within the 95% confidence interval. In contrast, COV displayed a highly skewed distribution, as depicted in Figure 1.

**Figure 2:**
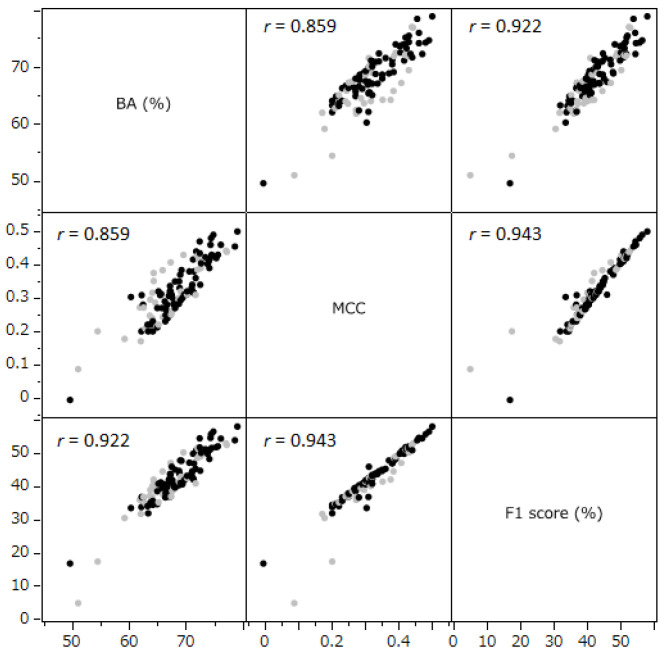
Correlation between the performance evaluation indicators for the predictive models. The analysis incorporated a total of 109 predictive models. Models with 100% coverage are represented in gray, while the other models are depicted in black. The terms BA (%) and MCC refer to balanced accuracy and Matthews correlation coefficient, respectively.

**Figure 3:**
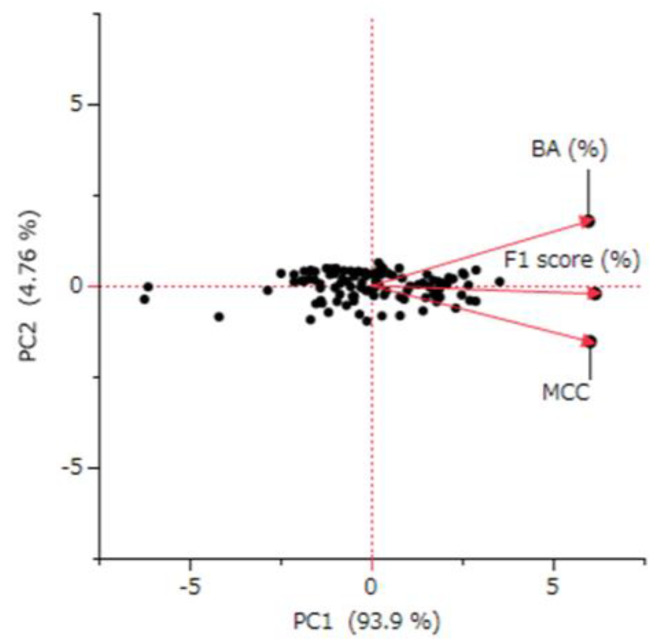
Principal component analysis of the performance evaluation indicators for the predictive models. The analysis incorporated a total of 109 predictive models. The figure displays a superimposed biplot of the score plot and loading vector. The terms BA (%), MCC, PC1, and PC2 refer to balanced accuracy, Matthews correlation coefficient, first principal component, and second principal component, respectively.

As depicted in Figure 2, we observed strong correlations between the BA, MCC, and F1 Score. The correlation coefficients were 0.859 between BA and MCC, 0.943 between MCC and F1, and 0.922 between F1 and BA. To derive a more integrated index from BA, MCC, and F1, we conducted a principal component analysis. The first principal component (PC1), which accounted for 93.9% of the variance, was selected as a comprehensive evaluation index (Figure 3). Therefore, PC1, along with BA, MCC, and F1, was used for subsequent analyses. However, it is important to note that the COV is determined by factors distinct from the model’s intrinsic performance. Therefore, COV was analyzed separately in correlation studies.

### 2.3. Evaluating the Impact of Coverage on Predictive Performance

A correlation analysis was conducted to assess the impact of the COV on BA, MCC, F1, and the PC1. Due to the skewed distribution of COV, Johnson normalization was applied to enable a normal quantitative correlation analysis [11,12].

Johnson Sb[COV] = ln[(COV − 4.9)/(100.063 − COV)] × 0.111 − 0.655

**Figure 4:**
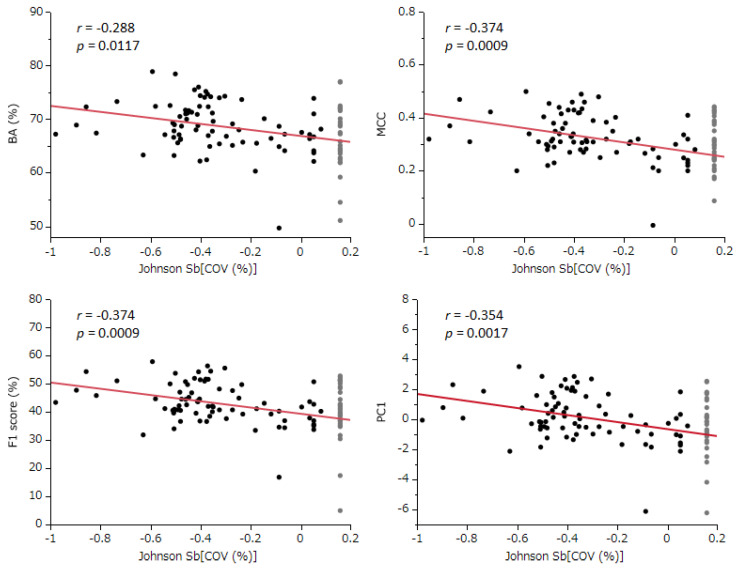
Correlation between the coverage rate and the performance evaluation index for predictive models. The terms BA (%), MCC, PC1, and Johnson Sb[COV (%)] represent the balanced accuracy, Matthews correlation coefficient, the first principal component, and Johnson normalized coverage, respectively. In the figure, prediction models with 100% coverage (33 species) are depicted in gray, while the other models (76 species) are shown in black. The analysis only includes prediction models with less than 100% coverage. The red line in the figure represents the least squares regression line, providing a visual representation of the correlation.

When models with 100% COV were excluded and the normal quantile plot was assessed, the Johnson Sb[COV] (normalized COV) demonstrated a normal distribution (Figure 1). A Pearson’s correlation analysis was conducted between this normalized COV (excluding 100% COV models) and BA, MCC, F1, and PC1. This analysis revealed significant negative linear correlations (Figure 2). The correlation coefficients for BA, MCC, F1, and PC1 were −0.288, −0.374, −0.374, and −0.354, respectively. The corresponding *p*-values were 0.0117, 0.0009, 0.0009, and 0.0017 (Figure 4). Regression diagnostics confirmed the normality of these least squares lines. These findings indicate that a lower coverage rate is associated with improved predictive performance.

### 2.4. Performance of All Models at 100% Coverage

In this study, we neutralized the influence of the COV and reassessed model performance. We used residuals from the linear relationship between normalized COV and each evaluation index to estimate the performance of all the models at 100% COV. The model with the highest BA of 76.9% at 100% COV was MMI-VOTE1, as reported by Meiji Pharmaceutical University (MPU). Additionally, the best predictive models for MCC, F1, and PC1 were all MMI-STK1, also reported by MPU. The respective values were 0.443 for MCC, 52.8% for F1, and 2.55 for PC1 (Table 1). Notably, all four top-ranking models, based on the comprehensive PC1 index, were developed by MPU, a first-time participant in the second challenge. Furthermore, the fifth and sixth most outstanding models were BM_PHARMA v1.5.2.0, submitted by MultiCASE Inc. (Mayfield Heights, OH, USA), and Derek_Nexus v.4.2.0, reported by Lhasa Limited (Leeds, UK) in the first project, respectively (Table 1).

### 2.5. Effect of Coverage on the Best Model

**Figure 5:**
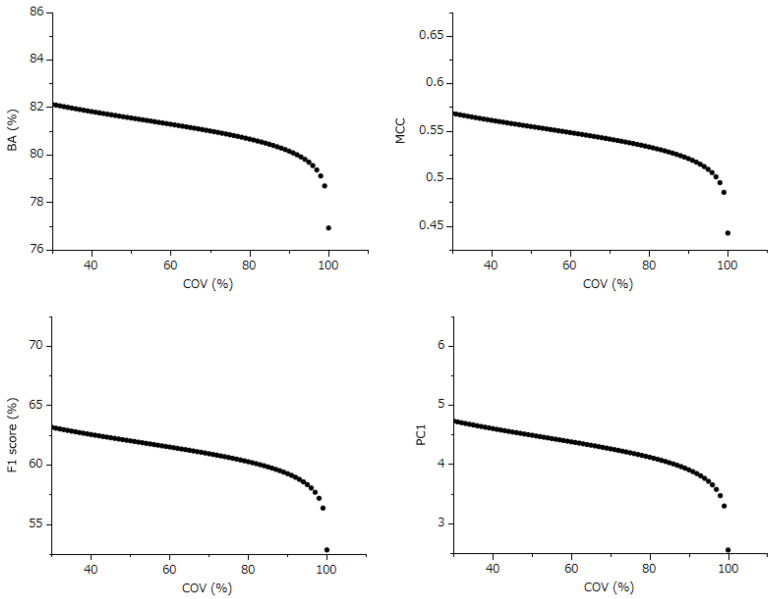
Estimation of performance evaluation index by coverage rate for the top prediction model. This figure illustrates the estimated variation in performance due to changes in the coverage rate of MMI-STK1 (Meiji Pharmaceutical University), which was identified as the highest-performing model based on the first principal component (PC1) as well as Matthews correlation coefficient (MCC) and F1 Score. The terms BA (%) and COV (%) denote the balanced accuracy and coverage rate for all predicted target compounds, respectively.

I estimated the performance changes in MMI-STK1, the top model in MCC, F1, and PC1, with varying COV. This estimation was achieved using the least squares line equation and residuals of predicted values in these models (Figure 4). For example, at COV levels of 100%, 90%, 60%, and 30% in this model, the F1 Score was estimated to be 52.8%, 59.2%, 61.5%, and 63.1%, respectively (Figure 5).

## 3. Discussion

### 3.1. Target Prediction Model for Reassessment

The first Ames/QSAR International Challenge Project [4], which was divided into three separate phase challenges, conducted each phase independently. Notably, the Ames test data for compounds used in the external validation sets of a previous phase were disclosed to participants before the start of the subsequent phase. This disclosure allowed participants to adjust their models using the newly available data. As a result, models that shared the same name across different phases might have been adjusted or modified. Furthermore, it was observed that the COVs were often reconfigured for models with the same name across various phases. Therefore, in this reanalysis, all the models used in each phase of the first project were treated as independent entities, regardless of whether they shared the same name.

On the other hand, during the second challenge [6], a rule was introduced that permitted participants to select which of their multiple submitted models would be evaluated. This rule aimed to reduce bias toward participants who submitted several models, thereby ensuring a fairer assessment. However, this approach also carried the risk of potentially high-performing models being excluded from the evaluation process. To mitigate this, in the current reanalysis, all the models submitted were extracted and included in the reassessment. As a result, the total number of models evaluated rose to 109 (Table S1).

### 3.2. Evaluation Index

Like many toxicity tests, the distribution of positive and negative compounds in the Ames test results is notably skewed. In the first Ames/QSAR International Challenge Project, the external validation set consisted of 12,140 compounds across three phases. Of these, 1757 (14.5%) were positive and 10,383 (85.5%) were negative. In the second challenge, the external validation set comprised 1589 compounds, with 236 (14.9%) testing positive and 1353 (85.1%) testing negative. It is widely acknowledged that using metrics such as sensitivity, specificity, accuracy, positive predictive value (PPV), and negative predictive value (NPV) for such imbalanced data can often lead to misleading evaluations [13,14].

Accuracy (Acc), while a frequently used evaluation metric, is notably vulnerable to skew in datasets that are imbalanced [15,16]. Acc is computed using the formula (TP + TN)/(TP + TN + FP + FN), where TP, TN, FP, and FN denote true positives, true negatives, false positives, and false negatives, respectively. This metric is straightforward and easy to comprehend. However, its reliability diminishes in scenarios like the project, where only 14.5% of the cases were positive. If all the test compounds were predicted to be negative, the resulting Acc would be 85.5%, which is misleadingly high. Consequently, a model that fails to accurately predict any positive cases may appear to outperform many others across both projects due to this metric’s susceptibility to data imbalance.

In predictive modeling, sensitivity (also known as recall) and specificity often display a trade-off relationship. This is also observed for PPV, also known as precision, which often has a complex relationship with sensitivity, depending on the threshold used in the model. Similarly, NPV and specificity also display a relationship that varies with different thresholds. To assess the robustness of a predictive model’s generalization performance, it is crucial to achieve a balanced mix of these metrics. As a result, receiver operating characteristic (ROC) curves and precision–recall curves are frequently utilized to examine these relationships [17,18]. The area under these curves is an excellent measure for model evaluation. However, these metrics are only applicable to statistical models that can calculate predicted probability values. They were not used in the Ames/QSAR international challenge projects due to their inability to evaluate knowledge-based models. Instead, balanced accuracy (BA), which is the average of sensitivity and specificity, was used as a comprehensive metric to evaluate these two parameters, replacing the ROC curve [19,20,21,22]. BA is calculated using a specific formula:

BA = (Sensitivity + Specificity)/2 = {TP/(TP + FN) + TN/(FP + TN)}/2

Indeed, the formula for BA combines sensitivity (the true positive rate) and specificity (the true negative rate), providing a single measure that encapsulates the model’s performance across both positive and negative cases in the dataset. BA is particularly insightful because it reflects the model’s accuracy in identifying classes, regardless of the size of each class in the sample. This makes BA an ideal metric for assessing the overall performance of a model, especially in situations where data classes are imbalanced. Furthermore, the interpretability of this indicator is excellent, offering a clear understanding of model accuracy in a balanced manner.

PPV, also known as precision, represents the proportion of items predicted as positive that are actually positive. On the other hand, sensitivity (or recall) signifies the proportion of actual positive items that are correctly identified as such. The F1 Score, which is the harmonic mean of PPV and sensitivity, is commonly used for a comprehensive evaluation of these two metrics [23,24]. It is important to note that sensitivity and PPV are also referred to as recall and precision, respectively. The F1 Score is calculated using the following formula:

F1 = 2 × Recall × Precision)/(Recall + Precision)

= 2 × (TP^2^)/{2 × (TP^2^) + TP × FP + TP × FN}

In the second Ames/QSAR International Challenge Project [6], the F1 Score was introduced as an additional evaluation metric, supplementing those used in the first project. The F1 Score is particularly beneficial in achieving a balance between PPV and sensitivity. A higher F1 Score indicates a superior model, as it represents a strong balance between predictive precision and recall. This is particularly important in scenarios such as Ames test predictions, where accurately detecting positives and minimizing false positives are equally important.

The MCC is another metric that is related to the chi-square statistic in a 2 × 2 contingency table. It incorporates a significant amount of information by considering the balance ratio of the four categories in the confusion matrix: TP, TN, FP, and FN [25,26]. The MCC is expressed as follows:

MCC = (TP × TN − FP × FN)/√{(TP + FP) × (TP + FN) × (TN + FP) × (TN + FN))}

Indeed, BA, the MCC, and the F1 Score each have their own unique statistical properties. However, they all serve as valuable integrative indicators for evaluating models, particularly when dealing with imbalanced data. In this study, a novel approach was adopted to streamline the performance evaluation of the predictive models. This approach involved combining these diverse indices using principal component analysis. This method offers a more consolidated and definitive evaluation metric, thereby simplifying the otherwise complex task of model assessment.

### 3.3. Principal Component Analysis

The principal component analysis (PCA) conducted in this study revealed a strong consolidation among BA, the MCC, and the F1 Score. These metrics accounted for 94% of the variance in the same principal component direction (Figure 3). This pattern suggests a significant collinearity among these metrics. Indeed, the correlation coefficients between these evaluation indicators showed strong correlations, ranging between 0.859 and 0.943 (Figure 2). This finding highlights the utility of the PC1, which integrates these metrics, as a common and definitive indicator of integrated predictive performance.

A crucial aspect of this study was the quantitative correlation analysis conducted to assess the impact of the COV on the evaluation indicators BA, MCC, F1, and PC1. Correlation analysis using Pearson’s correlation coefficient is generally most reliable when the variables under consideration follow a normal distribution [27]. However, when the variables deviate from normality, there is an increased risk of the analysis being influenced by outliers or an overestimation of the degree of correlation. Moreover, if the assumption of normal distribution is not met, the accuracy in determining significance levels may be compromised. Therefore, it is crucial to verify the normality of each dataset before performing correlation analysis. Upon checking the normal distribution of these parameters using normal quantile plots, a distribution heavily skewed toward the COV was observed. As a result, the Johnson normal distribution method [11,12] was employed to correct the COV distribution to a normal form, achieving effective normalization.

### 3.4. Impact of COV on Metrics

The conducted correlation analysis, which was between the normal COV and the comprehensive evaluation indicators BA, MCC, F1 Score, and PC1 unveiled statistically significant negative correlations across all metrics. This finding implies that models with better predictive performance are likely to have lower COV settings. Despite the fact that each participant uniquely determined the COV settings using various techniques, it is still possible to estimate the standard influence of COV across all models from the slope of the least squares line.

Following this, we used the slope of this linear relationship and the residuals from each evaluation index to estimate the values of these indices, assuming a COV of 100% for each model. This method effectively shifts the evaluation values of each model along the slope of the straight line to a point where the COV equals 100% (Figure 4). As a result, this calculation allows for a correction of all models to the evaluation indices at 100% COV, thereby enabling a fair comparison and evaluation across all models (Figure 4, and Table 1).

### 3.5. Best Models

The analysis, adjusted to a 100% COV for all models (Table 1 and Table S1), unveiled that the most efficacious predictive model, utilizing the integrated comprehensive index PC1, was MMI-STK1. This particular model, submitted by MPU during the second project, demonstrated superior performance. It is noteworthy that MMI-STK1’s training data exclusively encompassed the Phase 1, 2, and 3 datasets from the NIHS. For an in-depth understanding of the methodology contributing to its excellence, the algorithm employed for MMI-STK1 is elucidated in the supplementary file of the report [6]: “Ninety-nine stacking models were constructed using multiple descriptors (Dragon, MOE, Mordred) and various machine learning algorithms (light GBM, XG-Boost, deep learning, graph convolutional network). The ultimate prediction was determined through a majority vote from all prediction results. The descriptors utilized were computed through Dragon, MOE, and Mordred”.

My analysis identified MMI-VOTE1 as the second most effective model. Like MMI-STK1, MMI-VOTE1 was submitted by MPU during the second project and exclusively trained on NIHS-provided Phase 1, 2, and 3 data. The algorithmic approach for MMI-VOTE1 is extensively detailed in the supplementary file of the report [6]. This supplementary information provides a comprehensive insight into the methodologies and principles that underscore the success of MMI-VOTE1: “Nine stacking models were developed utilizing a combination of descriptors (Dragon, MOE, Mordred) and diverse machine learning algorithms (light GBM, XG-boost, deep learning, graph convolutional network). The final prediction was determined through a majority vote based on all prediction results. Descriptors were computed using Dragon, MOE, and Mordred”.

The third most effective predictive model identified in this study is MMI-STK2, submitted by MPU during the second project. In contrast to previous models, the training data for MMI-STK2 included not only the Phase 1, 2, and 3 data provided by the NIHS but also Hansen’s data. The detailed algorithmic approach for MMI-STK2 is expounded upon in the supplementary file of the report [6]. “MMI-STK2 is a stacking model constructed using Light GBM, deep learning, and graph convolutional network algorithms, with descriptors calculated using Dragon and MOE”.

The fourth best-performing model identified in the analysis was MMI-VOTE2, another model submitted by MPU during the second project. Similar to MMI-STK2, the training data for MMI-VOTE2 not only included the Phase 1, 2, and 3 data from the NIHS, but also incorporated Hansen’s data. More detailed information about the algorithm used for MMI-VOTE2, including its approach to integrating these diverse datasets, is available in the supplementary file [6]: “MMI-VOTE2 is a majority voting model constructed using Light GBM, Deep Learning, Random Forest, and graph convolutional network algorithms. The descriptors used were calculated using Dragon, MOE, and DNA docking simulations”.

In this project, MPU registered the four types of models previously mentioned. Impressively, all these models ranked as the top performers among the 109 models evaluated in this study. These models did not undergo adjustments to their coverage rate by altering their applicability domain. Given that setting a model’s applicability domain is a highly technical process and varies significantly from model to model, it is reasonable to infer that the estimated values of the metrics used for evaluating prediction models at COV levels other than 100% might contain considerable errors. Despite this, the results mentioned above demonstrate the current technical capabilities of Ames prediction, underlining both the advances and potential limitations in this field.

### 3.6. Effect of Coverage on the Best Model

For MMI-STK1, which was identified as the top-performing model in terms of MCC, F1, and the integrated index PC1, we estimated how BA, MCC, F1, and PC1 would change with alterations in the COV (refer to Figure 5). For example, while the F1 Score was at 52.8% with a COV of 100%, it was projected to increase to 59.2%, 61.5%, and 63.1% when the COV was adjusted to 90%, 60%, and 30%, respectively. This trend indicates that the predictive performance of the model can significantly improve even with a 90% COV, which involves excluding only 10% of the compounds from the prediction. This observation implies that the external validation set included a small proportion of compounds that this model found particularly challenging to predict accurately.

These results lead to an important conclusion: when applying the Ames prediction model in real-world scenarios, it is crucial to consider the applicability domain while setting the COV [7,8,9,10]. Although the estimated values presented here might contain substantial errors, the performance of the model could be further improved if the COV settings are based on appropriately defined applicability domains.

### 3.7. Evaluation of Adaptive Domain Setting Technology and Future Prospects

This study represents the first instance in the Ames/QSAR Challenge Projects where it has been explicitly shown that the performance of predictive models can be improved by adjusting COV settings. Notably, it also pinpointed the model with the highest predictive performance by taking into account COV. This significant discovery, achieved in a highly competitive environment, highlights the current limitations of QSAR technology in predicting Ames test outcomes. However, the enhanced prediction performance attributed to COV settings depends on the accurate definition of the models’ applicability domain. The performance evaluation at 100% COV presented in this study is essentially a projection based on standard COV settings. With careful consideration of the applicability domain settings, there is potential to exceed these standard performance levels.

While the technology for setting applicability domains—evaluated based on compound similarities and predicted probabilities derived from the models—is advanced, the research on optimal methodologies for setting applicability domains is still in its early stages [7,8,9,10]. This reanalysis had limited capacity to assess this specific aspect of model performance. However, as technologies for systematically determining suitable applicability domains for each model advance, a combination of diverse models, like those presented in this project, could lead to improved prediction accuracy. Although this project was primarily a competition evaluating the standalone performance of various models, future enhancements in prediction rates are expected, especially with the application of ensemble and consensus methods based on advanced techniques for estimating applicability domains [28].

## 4. Methods

### 4.1. Analysis Strategy

The overarching strategy for this reanalysis is outlined in the following steps:

a. Model and value extraction: retrieve all prediction models and their corresponding evaluation values from the Ames/QSAR 1st and 2nd Challenges.

b-1. PCA: perform PCA using balanced accuracy (BA), Matthews correlation coefficient (MCC), and F1 Score to calculate the first principal component (PC1) as a comprehensive evaluation index.

b-2. Normalization of COV distribution: normalize the distribution shape of the compound sample COV.

c. Correlation analysis: conduct a thorough correlation analysis between BA, MCC, F1, and PC1 against COV, and derive the least squares regression line.

d. Estimation at 100% COV: estimate the predictive performance of all models at a COV of 100% using the least squares regression line and considering the residuals of each evaluation index.

e. Identifying the best model at 100% COV: evaluate and determine the best predictive model at a COV of 100%.

f. Performance estimation with varying COV: estimate the predictive performance of the best model across various COV settings.

### 4.2. Data for the Analysis

The analysis encompassed all prediction models and their associated evaluation metrics as documented in both the main texts and supplementary materials of the 1st and 2nd Ames/QSAR International Challenge Projects papers [4,6]. In the context of the first challenge, any prediction models sharing the same name but employed in different phases were treated as distinct models for evaluation purposes. This approach acknowledges the potential modifications and adjustments made to the models across various phases. In the second project, the analysis considered all prediction models listed in the supplementary file of the project paper. This comprehensive approach ensured that all the models submitted for the challenge were taken into account in the analysis.

### 4.3. Evaluation Index

In the Ames/QSAR International Challenge Projects, compounds underwent categorization into three classes based on the Ames test results [4,6]:

Class A (strong positive): compounds inducing over 1000 revertant colonies per milligram in at least one Ames test strain, with or without metabolic activation.

Class B (positive): these compounds caused a minimum 2-fold increase in revertant colonies compared with the negative control, but less than those induced by Class A compounds, in at least one Ames strain with or without metabolic activation.

Class C (negative): defined as compounds indicating less than a 2-fold increase in revertant colonies (non-mutagenic).

Challenge participants were tasked with submitting results identifying positive compounds (Classes A and B) and negative compounds (Class C) through either existing or newly developed predictive models. The organizers then computed various metrics, including sensitivity for Class A, sensitivity, specificity, accuracy, BA, MCC, and F1 Score, based on these predictions. As mentioned above, since sensitivity and specificity are in a trade-off relationship, observing only one evaluation index might lead to incorrect interpretations [17,18,19,20,21,22]. In addition, accuracy is reportedly an inappropriate imbalanced data evaluation indicator [15,16]. Therefore, in this study, BA, MCC, and F1 were chosen as comprehensive evaluation metrics for generalization performance and used for analysis. It is important to note that the F1 Score was not used as an evaluation metric in the first challenge. To ensure consistency, the F1 Scores for all models in the first project were retrospectively calculated from the PPV (precision) and sensitivity (recall), following the method used in the second project (Table S1). The normality of the comprehensive evaluation indicators (BA, MCC, F1) and the coverage ratio (COV) was assessed using normal quantile plots and their respective 95% confidence intervals. Subsequently, the evaluation indicators (BA, MCC, F1) underwent PCA to calculate the PC1. In addition to these measures, this study also focused on evaluating the impact of COV fluctuations on model performance.

### 4.4. Evaluating the Impact of Coverage on Predictive Performance

The COV was initially normalized using the Johnson normalization method [11,12]. Following this, a correlation analysis was performed between the normalized COV and the comprehensive evaluation indicators: BA, MCC, F1 Score, and the PC1. This analysis did not include models that were already reported to have a 100% COV. Based on the results of this correlation analysis, especially the residuals from the least squares regression line, this study estimated the evaluation indices of all predictive models at a COV of 100%. In addition, this study explored estimating the predictive performance of the model identified as the best under different COV conditions. Specifically, the evaluation index for each model at 100% COV was calculated from the equation of the least squares line between the normally distributed COV and BA, MCC, F1, or PC1, and the residuals of each model from those lines.

### 4.5. Statistical Test

The normality of each evaluation index was confirmed by examining the 95% confidence intervals illustrated in normal quantile plots. This step ensured that the data adhered to the assumptions necessary for subsequent statistical analyses. Pearson’s correlation coefficient was utilized to assess the relationship between various evaluation indices. A significance level of 0.05 was established to determine the statistical significance of the correlations, following standard practices in statistical testing. PCA was carried out using a correlation coefficient matrix. All statistical analyses were conducted using JMP Pro version 16.2, software developed by SAS Institute Inc., Cary, NC, USA.

## 5. Conclusions

This study is the first in the Ames/QSAR Challenge Projects to demonstrate that the performance of predictive models can be improved by adjusting the COV. It emphasizes a model with exceptional predictive performance when considering COV, thereby showcasing the highest current QSAR technology level in predicting Ames test outcomes. This study suggests that with accurate settings of the applicability domain, model performance could see improvements. While the existing technology for setting applicability domains is sophisticated, research in this field is still in its early stages. This study could point out the potential for enhanced prediction accuracy through the integration of diverse models and anticipates future advancements with the application of ensemble and consensus methods, particularly in the estimation of applicability domains.

## Figures and Tables

**Table 1:** Top 20 prediction models at 100% coverage rate.

Project No	Teams	QSAR Tools (Module)	Estimated BA (%)	Estimated MCC	Estimated F1 Score (%)	Estimated PC1
2	Meiji Pharmaceutical University	MMI-STK1	76.9	0.44	52.8	2.55
2	Meiji Pharmaceutical University	MMI-VOTE1	77.0	0.44	52.4	2.51
2	Meiji Pharmaceutical University	MMI-STK2	72.0	0.43	51.6	1.82
2	Meiji Pharmaceutical University	MMI-VOTE2	72.0	0.43	51.4	1.78
1	MultiCASE Inc.	BM_PHARMA v1.5.2.0 (Statistical approach; SALM/ECOLI consensus)	74.7	0.40	49.6	1.76
1	Lhasa Limited	Derek_Nexus v.4.2.0	72.2	0.42	51.2	1.75
2	Lhasa Limited	Derek Nexus v.6.0.1	72.1	0.42	51.0	1.72
1	Swedish Toxicology Science Research Center	Swetox AZAMES_2	71.7	0.42	50.5	1.63
1	Molecular Networks GmbH and Altamira LLC	ChemTunes•ToxGPS Ames (original)	71.7	0.42	50.5	1.62
1	Prous Institute	Symmetry S. typhimurium (Ames)_2	73.3	0.40	49.6	1.59
2	NIBIOHN	GNN(kMoL)_bestF1	69.5	0.43	50.1	1.42
1	Lhasa Limited	Derek_Nexus v.4.0.5	72.5	0.39	48.9	1.42
1	Leadscope Inc.	Statistical-based QSAR (rebuild I)	72.8	0.38	48.0	1.34
2	MN-AM	ChemTunes.ToxGPS Ames NIHS_v2	74.8	0.37	46.4	1.33
2	Evergreen AI, Inc.	Avalon	71.9	0.38	48.5	1.29
1	Leadscope Inc.	Rule-based (Alerts)	71.3	0.39	48.8	1.27
1	MultiCASE Inc.	GT_EXPERT v1.5.2.0 (Rule based)_2	72.2	0.35	45.5	0.88
1	Molecular Networks GmbH and Altamira LLC	ChemTunes•ToxGPS Ames (enhanced)_1	72.2	0.35	45.7	0.87
2	NIBIOHN	GNN(kMoL)_bestbalanced (the best model)	67.2	0.41	47.0	0.79
1	Swedish Toxicology Science Research Center	SwetoxAZAMES v2	71.3	0.35	45.2	0.77

This table lists the top 20 prediction models performing best at a 100% coverage rate, as estimated by the first principal component (PC1). The terms BA (%), MCC, and PC1 refer to balanced accuracy, Matthews correlation coefficient, and first principal component, respectively. The higher the performance in each evaluation metric, the darker the red color shown.

## Data Availability

All data used in this study have been published in their available form in the following two papers: https://doi.org/10.1093/mutage/gey031, https://doi.org/10.1080/1062936X.2023.2284902.

## Supplementary Materials

The following supporting information can be downloaded at: https://www.mdpi.com/article/10.3390/ijms25031373/s1.
